# Pectin/Gellan Gum Hydrogels Loaded with *Crocus sativus* Tepal Extract for In Situ Modulation of Pro-Inflammatory Pathways Affecting Wound Healing

**DOI:** 10.3390/polym17060814

**Published:** 2025-03-20

**Authors:** Francesco Busto, Caterina Licini, Stefania Cometa, Stefano Liotino, Elisabetta Damiani, Tiziana Bacchetti, Isabelle Kleider, Alessandra La Contana, Monica Mattioli-Belmonte, Elvira De Giglio

**Affiliations:** 1 Department of Chemistry, University of Bari, Via Orabona 4, 70126 Bari, Italy; francesco.busto@uniba.it (F.B.); stefano.liotino@uniba.it (S.L.); 2 INSTM, National Consortium of Materials Science and Technology, Via G. Giusti 9, 50121 Florence, Italy; 3 Department of Clinica and Molecular Science, Università Politecnica delle Marche, via Tronto 10/a, 60126 Ancona, Italy; c.licini@staff.univpm.it (C.L.); a.lacontana@pm.univpm.it (A.L.C.); m.mattioli@staff.univpm.it (M.M.-B.); 4 Jaber Innovation s.r.l., Via Calcutta 8, 00144 Rome, Italy; 5 Department of Life and Environmental Sciences, Polytechnic University of Marche, 60131 Ancona, Italy; e.damiani@staff.univpm.it (E.D.); t.bacchetti@staff.univpm.it (T.B.); 6 Anton Paar TriTec SA, Vernets 6, 2035 Corcelles, Switzerland; isabelle.kleider@anton-paar.com; 7 Advanced Technology Center for Aging Research, IRCCS INRCA, Via Birarelli, 60121 Ancona, Italy

**Keywords:** *Crocus sativus* tepal extract, pectin, gellan gum, antioxidant properties, wound healing

## Abstract

Tepals of the *Crocus sativus* flower constitute the most abundant floral residue during saffron production (350 kg tepals/kg stigmas). Being a natural source of polyphenols with antioxidant properties, they can be reused to create potentially valuable products for pharmaceutical applications, generating a new income source while reducing agricultural bio-waste. In this work, composite hydrogels based on blends of pectin and gellan gum containing *Crocus sativus* tepal extract (CSE) have been proposed for the regeneration and healing of cutaneous wounds, exploiting the antioxidant properties of CSE. Various physico-chemical and mechanical characterizations were performed. The skin permeation of CSE was investigated using Franz cell diffusion system. The composite films were cytocompatible and able to counteract the increase in ROS, restore the production of matrix proteins, and favor wound closure. To conclude, CSE-loaded composite films represent a promising strategy to promote the body’s natural healing process. In addition, by reusing saffron tepals, not only can we develop new, sustainable treatments for skin diseases, but we can also reduce agricultural waste.

## 1. Introduction

Wounds resulting from diseases such as diabetes, accidents, or intentional actions present significant medical challenges because of the intricate nature of the healing mechanism [1]. Indeed, imperfect wound healing has an impact on both recovery times and healthcare expenses, where it is estimated that over $250 billion is spent globally each year to tackle this problem, and that around 5 million people die annually because of poorly managed wounds [2]. Recent advancements in wound healing have introduced several innovative approaches, that are paving the way for more effective and efficient wound care therapies. These include the use of nanomaterials [3], 3D-printed biomaterials [4], stem cell therapy [5], growth factors and cytokines [6,7], and bioactive dressings and hydrogels [8], the latter being the motivation of the present study. Advanced wound dressings that incorporate hydrogels and bioactive compounds provide a moist environment and deliver therapeutic agents directly to the wound site aiding the healing process. Among these agents are plant-based products that have been consistently used throughout human evolution to treat skin damage, aging diseases, and heal wounds [9,10,11]. They contain various secondary metabolites like phenolic compounds, steroids, flavonoids, and more, which aid the wound healing process through anti-inflammatory, antioxidant, antimicrobial, and other mechanisms [9]. Examples include extracts from turmeric [12], aloe vera [13], tea tree and neem [14], calendula [15], *Centella asiatica* [16], lavender, and chamomile [17,18]. Building on the well-documented benefits of traditional plant-based products, this study explores the untapped potential of *Crocus sativus* flower tepals (CSE) as a sustainable and effective source of bioactives for wound healing hydrogels. Traditionally, the focus has mainly been on the stigmas of *C. sativus* commonly identified as the spice saffron, known for its culinary and medicinal uses. Its high content in flavonoids and the carotenoids safranal, crocin, and crocetin contribute to the strong antioxidant, anti-inflammatory, antimicrobial activities crucial for the wound healing properties [19,20,21,22]. However, during saffron production, a huge amount of tepals is discarded as waste, which could be exploited to develop new sustainable treatments for wound management. Indeed, CSE possesses significant bioactive properties prompting investigations as a potential natural remedy for accelerating cutaneous wound repair [23,24,25].

Soheilifar et al. reported that CSE promoted cell viability, migration, and angiogenesis, and enhanced collagen synthesis and wound closure in diabetic mice, suggesting its potential as a treatment for diabetic wounds [26]. Similar findings were reported by Verjee et al., who showed that CSE promoted scratch wound closure and vascular endothelial growth factor secretion in keratinocytes [27]. However, the use of CSE for health products directed at wound dressings is scarce. To the best of our knowledge, only two studies have been reported on the use of CSE drug-delivery systems for this purpose: Pagano et al. proposed a CSE–starch gel formulation to spread on damaged skin, while Zeka et al. used hydrogels composed of two biocompatible, synthetic polymers, i.e., polyvinyl pyrrolidone and polyethylene glycol, to deliver antioxidant compounds isolated from CSE [28,29]. In the present study, to valorize CSE within the circular economy framework, we developed hydrogel films for wound healing applications by incorporating CSE into blends of two natural, biodegradable and biocompatible polysaccharide polymers: pectin (Pec) and gellan gum (GG).

Pec, a natural macromolecular polysaccharide mainly present in cell walls of higher plants and in fruit peels, is widely acknowledged as a promising biopolymer for producing packaging films, due to its exceptional film-forming capabilities, biodegradability, and biocompatibility [30]. When Pec is employed in wound dressing, it allows wound exudate removal and provides an acidic environment to avoid microbial growth encouraging the wound-healing process [31]. However, the applications of Pec films are limited due to their poor mechanical properties and high hydrophilicity compared with conventional films [32]. To overcome this challenge, blends of Pec with other biopolymers and/or plasticizers, together with the use of crosslinking agents, have been developed to enhance pectin films’ physical properties [33,34].

GG is a natural anionic polysaccharide, obtained from the fermentation of *Pseudomonas elodea*, interesting for wound healing due to its high flexibility, elasticity, and moist conditions suitable for skin diseases [35,36,37]. To the best of our knowledge, a blend based on Pec and GG for biomedical applications was previously proposed only by Prezotti et al., who exploited the polymers mucoadhesive properties to obtain an oral drug delivery system [38]. Here, to improve the mechanical properties of the Pec-GG films, tartaric acid was chosen as an easily accessible, cost-effective, and green crosslinker [39] while glycerol was used as plasticizer. Moreover, a further crosslinking step using calcium ions was employed to enhance the hydrogel film stability.

A detailed physicochemical characterization of the Pec-GG/CSE hydrogel films was carried out by spectroscopic (FT-IR/ATR, XPS), thermal (TGA and DSC), and mechanical (indentation test) techniques. In vitro antioxidant activity and the total polyphenol content of the CSE and CSE-loaded hydrogels were also evaluated. In vitro skin permeation studies by Franz cell allowed assessment of the transdermal delivery of the CSE components. Liquid uptake studies evidenced the swelling capability of carbohydrate-based films, also when loaded with CSE. For biological assessments, we focused on several aspects related to the wound healing process, from the antioxidant properties to the healing ability of the films. With this purpose, the developed films were tested on normal and damaged fibroblasts. In the latter, we observed a reduction in intracellular ROS and an increase in antioxidant enzymes, as well as restored morphology and migrative capability after treatments.

By incorporating CSE into novel hydrogels made from the combination of bio-based, biodegradable, and biocompatible polymers, Pec-GG, which have not been previously proposed for wound healing purposes, the study provides a step forward in addressing several gaps in current research on plant-based products for wound healing. These include understanding of the precise mechanisms through which active compounds exert therapeutic effects [40], and integration with modern therapies, for instance, innovative hydrogels. Addressing these gaps will help advance the field of wound healing and improve patient outcomes.

## 2. Materials and Methods

### 2.1. Materials

Pectin (viscosity 30.5 to 36.5, degree of esterification 70%, Cat. 416862500, Thermo Scientific, Waltham, MA, USA)—coded as Pec—and low-acyl content gellan gum (Phytagel™, molecular weight 1 × 10^6^ g/mol)—coded as GG—were purchased from Sigma-Aldrich (Milan, Italy). Tartaric acid, calcium chloride and glycerol were purchased from Sigma-Aldrich (Milan, Italy). *Crocus sativus* tepals were donated by a local farm in the Marche region. The lyophilized *Crocus sativus* tepal aqueous extract—coded as CSE—was obtained and characterized as previously described in Bellachioma et al. [24] with minor modifications. In the present study, 2 g of dried tepals, instead of 1 g, were stirred overnight at 4 °C in 100 mL water, to obtain a more concentrated aqueous extract. After incubation, the samples were centrifuged at 1000× *g* for 10 min and the collected supernatants were filtered (12 µm Albet filter paper). In the present study, CSE was then lyophilized to increase its shelf life and to facilitate its incorporation into the hydrogel films. Hydrogel films were prepared with ultrapure water, obtained through a Milli-Q^®^ distillation system (Millipore-Merck, Darmstadt, Germany). All solvents and reagents were purchased from Sigma Aldrich (Milan, Italy), unless otherwise specified.

### 2.2. Hydrogel Films Preparation

The formulation of the films was optimized by testing different GG/Pec ratios, maintaining the sum of the two polymers percentages equal to 2% (*w*/*v*), in agreement with our previous studies relevant to gellan gum and sodium alginate blends for skin care applications [37]. Three different GG:Pec ratios were tested: GG 1.6% and Pec 0.4% *w*/*v* (GG_1.6_-Pec_0.4_), GG 1.0% and Pec 1.0% *w*/*v* (GG_1.0_-Pec_1.0_), and GG 0.4% and Pec 1.6% *w*/*v* (GG_0.4_-Pec_1.6_). Tartaric acid at 0.2% (*w*/*v*) was used as crosslinker and initially solubilized in distilled water at 80 °C, followed by the polymer powder addition under continuous stirring on a magnetic plate (VELP Scientifica Srl, Usmate, Italy). Glycerol at 1% *w*/*v* was finally added to the solution as plasticizer. When CSE-loaded films were produced, 1.6 mg/mL of CSE were added to the solution as the final step, after lowering the temperature to 40 °C to avoid bioactive molecules degradation. The appropriate CSE amount to load in the hydrogel films was chosen according to viability assay experiments described by Bellachioma et al. [41]. Smooth and homogeneous film-forming solutions were cast on Petri dishes (Corning Inc., Corning, NY, USA), 9 cm diameter, and dried in an oven at 37 °C for 48 h. Lastly, the films were dipped for 15 min in 5% *w*/*v* CaCl_2_ ethanolic solution, inducing a second crosslinking step, to obtain less swellable and more homogeneous hydrogel films. Blank films, without CSE, were also prepared as control to highlight the role of CSE in films’ bioactive performances. In Table 1, a summary of the different hydrogel films prepared is reported.

### 2.3. Physico-Chemical Characterization

#### 2.3.1. X-Ray Photoelectron Spectroscopy (XPS)

The prepared dried films were examined by means of XPS (PHI 5000 VersaProbe II, Chanhassen, MN, USA). Analyses were performed in high power mode with an AlKα X-ray radiation source and an instrument base pressure of 10^−9^ mbar. Wide scans and high-resolution scans were recorded in fixed analyzer transmission (pass energy of 117.4 eV and 29.35 eV, respectively). MultiPak software (v.9.9.0.8) was used for data mining, setting the reference charge at 284.8 eV (hydrocarbon peak).

#### 2.3.2. Fourier Transform Infrared Spectroscopy (FT-IR) in Attenuated Total Reflectance Mode (ATR)

Dried samples were analyzed by means of FT-IR (ATR) analyses through a Spectrum Two PE instrument supplied by PerkinElmer (Waltham, MA, USA), endowed with a universal ATR accessory (UATR, Single Reflection Diamond/ZnSe). For each of the relevant samples, FT-IR/ATR spectra were recorded from 400 to 4000 cm^−1^ with a 4 cm^−1^ resolution.

#### 2.3.3. Thermo-Gravimetric Analysis (TGA)

The thermal behavior of the dehydrated hydrogels was assessed on a PerkinElmer TGA-400 instrument (Perkin Elmer, Milan, Italy), heating 5–10 mg of the samples in the range 30–600 °C. The analyses were performed in nitrogen, with a gas flow set at 20 mL/min. Data were recorded by means of the TGA Pyris software (version 13.3.1.0014).

#### 2.3.4. DSC

Thermal properties were investigated on a Perkin Elmer (Waltham, MA, USA)—DSC 4000 calorimeter. The nitrogen flow was 20 mL/min at a heating rate of 10 °C/min. The samples were heated from 30 °C to a maximum temperature of 180 °C. Samples of about 2 mg were used for DSC measurements.

### 2.4. Evaluation of Antioxidant Activity by ABTS and DPPH Assays

The antioxidant activity of CSE and CSE-loaded films was tested using the ABTS and DPPH assays, as described by Luo et al. [42], with some modifications [37,43]. ABTS was dissolved in PBS (pH 7.4) at a concentration of 7 mM with 2.45 mM of ammonium persulfate (APS). The radical cation (ABTS˙+) was obtained after 16 h of reaction in the dark and then diluted to reach an absorbance of 0.70 ± 0.02 at 734 nm before use. An amount of 0.2 mL of the sample (in the range 1–1000 μg/mL) was mixed with 2.0 mL of ABTS˙+ and the absorbance was measured at 734 nm after 6 min. The radical scavenging activity percentages (%RSA) were calculated using the following equation:

%RSA = [A_0_ − (A_s_ − A_b_)]/A_0_ × 100

where A_0_ is the ABTS˙+ absorbance, As is the absorbance of ABTS˙+ with the sample, and A_b_ is the absorbance of the sample without the radical cation.

In the DPPH assay, the DPPH radical was dissolved in methanol at a concentration of 100 µM and its absorbance was measured at 517 nm. Simultaneously, different aqueous solutions of the sample (50–1000 µg/mL) were prepared. Sample solution (3 mL) was mixed with 1 mL DPPH solution and the absorbance was measured at 517 nm. The % RSA were calculated with the following equation:

%RSA = (A_rad_ − A_S_)/A_rad_ × 100

in which A_S_ represents the sample’s absorbance, whereas A_rad_ is the absorbance of the DPPH radical alone. Each point was performed in triplicate and expressed as mean ± standard deviation. All the assays were performed using a UV-visible Spectrophotometer UV-1900i (Shimadzu, Milan, Italy). For both assays, the antioxidant activity of each CSE-loaded film was evaluated and compared with CSE aqueous samples having a concentration equal to that loaded in the film. Each film (1 × 1 cm^2^) was immersed 24 h in PBS, and the resulting solution was tested with the ABTS and DPPH assays.

### 2.5. Total Polyphenol Content (TPC)

The TPC in CSE and CSE-loaded films was determined using the Folin–Ciocâlteu colorimetric method as described by Fabiano et al. [44] with some modifications [37]. Briefly, 0.5 g of gallic acid (GA) were dissolved in 10 mL ethanol and subsequently brought up to 100 mL volume with distilled water, obtaining a concentration of 5 g/L. Afterwards, 100, 150, 250, and 500 mg/L standard solutions from GA stock solution were obtained. An amount of 100 μL of each GA solution was added to 7.9 mL of distilled water and 500 µL of Folin–Ciocâlteu reagent. After 8 min, 1.5 mL of sodium carbonate solution (1.9 M) were added, and the absorbance was measured spectrophotometrically using a wavelength range from 690 to 810 nm and measuring the absorbance value against the blank at a wavelength of 765 nm using a UV-visible Spectrophotometer UV-1900i (Shimadzu, Milan, Italy). A triplicate of each sample was performed. The TPC in CSE was therefore determined using the calibration curve, and the results are expressed as mg of gallic acid equivalent per g of dry material, i.e., mg GAE/g. The calibration equation for GA was y = 0.0011x − 0.009 (R^2^ = 0.9999). To evaluate the polyphenols released from the CSE-loaded films, 1 cm^2^ of each film (average mass of 10.0 ± 0.5 mg) was dipped in 5 mL PBS for 24 h at 25 °C and the resulting solution was analyzed to obtain the TPC.

### 2.6. In Vitro Skin Permeation Studies

The in vitro skin permeation studies were performed using a Franz diffusion cell (PermeGear Inc., SES GmbH, Bechenheim, Germany) using a previously reported protocol [37]. Briefly, CSE-loaded films (about 1 cm^2^) were placed in the cell donor compartment. An O-ring joint kept the film on the synthetic StratM^®^ membrane (Merck KGaA, Darmstadt, Germany), characterized by skin-like porosity, diffusivity and composition. The whole assembly was fixed with a stainless-steel clamp to maintain the tight connection between the donor and receptor compartments. Phosphate buffer (PBS) (pH 7.4) solution (1 mL and 5 mL) were added in the donor and acceptor compartments, respectively. In the acceptor compartment the buffer solution was continuously stirred using an ATE magnetic stirrer (VELP Scientifica Srl, Usmate, Italy). The temperature was kept constant at 32.00 ± 0.03 °C using a CD-B5 heating circulator bath (Julabo GmbH, Seelbach, Germany). To evaluate the kinetic release of polyphenols, at predetermined time points (30 min, 1, 2, 8, and 24 h), PBS aliquots of 500 μL were withdrawn, replaced with fresh buffer, and the polyphenol content was calculated as described in the previous section. At the end of the experiment, the StratM^®^ was sectioned with a scalpel into six parts and placed into 5 mL of PBS, vortexed (VELP Scientifica Srl, Usmate, Italy) for 1 min and left 24 h at 25 °C to extract and quantify the TPC retained by the membrane [37,42].

### 2.7. Hydrogel Films Swelling and Water Holding Capacity

Dry hydrogel samples were cut into square pieces with 1 cm^2^ area, accurately weighed (m_id_) and immersed in phosphate-buffered solution (PBS) or simulated wound fluid (SWF) at 32 °C to determine the swelling kinetics up to 24 h. All the specimens were weighed after each time point (m_it_). The water uptake (expressed as g of PBS or SWF per g of dry film) was calculated using the following formula:

Water uptake = (m_it_ − m_id_)/m_id_

Moreover, for water holding capacity measurements, the hydrated hydrogel films were centrifuged in a centrifuge equipped with a basket rotor at1400 rpm. The centrifuged samples were weighed, and the retained water was expressed as a weight percentage to the total water uptake:

Water Holding Capacity = (m_c_ − m_d_)/(m_s_ − m_d_) × 100

where m_c_, m_s_, and m_d_ are the weights of centrifuged, swollen, and dry hydrogels, respectively.

### 2.8. Indentation Test

Hydrogel film samples were adhered to a petri dish using a thin layer of cyanoacrylate glue and immersed in water. Instrumented indentation measurements were performed on the different hydrogels using a Bioindenter nanoindentation device (Anton Paar Tritec, Corcelles, Switzerland). A ruby spherical indenter with a radius of 500 µm was employed to penetrate the hydrogels under an incrementally applied normal load. Testing parameters were adapted for the distinct properties of each sample (softer samples required lower load and vice versa) and to measure elastic modulus (10 s pause at max. load) and creep properties (60 s hold at max. load) (Table 2).

The applied load was recorded as a function of the corresponding indenter displacement in the hydrogel (indentation depth), enabling the calculation of the elastic modulus and creep behavior using the Oliver and Pharr models. The indentation modulus E_IT_ was obtained from the formula:

$$\frac{1}{E_{r}}=\frac{1-{n_{s}}^{2}}{E_{IT}}+\frac{1-{n_{i}}^{2}}{E_{I}}$$

Here, n_i_ represents Poisson’s ratio of the ruby spherical indenter, n_s_ is Poisson’s ratio of the hydrogel sample, E_i_ is the modulus of the indenter, and E_r_ is the reduced modulus given by:

$$E_{r}=\frac{\sqrt{\pi }\cdot S}{2\cdot \beta \cdot \sqrt{A_{p}(h_{c})}}$$

where S is the contact stiffness, A_p_(h_c_) is the projected contact area of the indenter, and β is a geometric factor (1.0 for spherical indenter).

The indentation creep C_IT_ was measured during the 60 s pause and calculated with the following formula:

$$C_{IT}=\frac{h_{2}-h_{1}}{h_{1}}$$

where h_1_ is the indentation depth at the start of the pause and h_2_ is the indentation depth at the end of the pause. This parameter provides a quantitative measure of the time-dependent deformation under constant load, capturing the viscoelastic behavior of the hydrogel samples.

### 2.9. Biological Evaluation

#### 2.9.1. Cells and Conditioned Media Production

Normal human dermal fibroblasts (NhDF) were cultured in High Glucose Dulbecco’s Modified Eagle Medium (HG-DMEM; Corning Inc., Corning, NY, USA), supplemented with 10% fetal bovine serum (Corning Inc.), 1% L-glutamine (Thermo Fisher Scientific, Waltham, MA, USA) and 1% penicillin/streptomycin (Thermo Fisher Scientific) and incubated at 37 °C with 5% CO_2_. For all the experiments, NhDFs were seeded at 7500/cm^2^. To obtain the conditioned media (CM), films were incubated for 24 h with medium (1 cm^2^/mL). To simulate oxidative stress conditions, cells were treated with 200 μM H_2_O_2_ for 24 h, before incubation with CM. Controls are represented by cells cultured in normal medium (NM).

#### 2.9.2. Film Cytocompatibility

To assess films’ cytocompatibility, NhDFs were seeded and treated with CM for 24 h. Cell viability was then evaluated with the Alamar Blue assay (Invitrogen, Waltham, MA, USA) according to the manufacturer’s instruction. The emitted fluorescence was detected on a microplate reader Infinite 200 PRO (Tecan, Männedorf, Switzerland) with excitation/emission at 535/590 nm.

#### 2.9.3. Morphological Observation and Stress Fiber Evaluation

Cells were fixed with 4% paraformaldehyde in PBS pH 7.4 at 4 °C for 30 min, washed in PBS three times, and permeabilized with 0.1% Triton X-100 in PBS at RT for 30 min. To highlight cytoskeletal fibers, cells were then incubated with TRITC-labelled Phalloidin (Thermo Fisher Scientific, dil. 1:1000) for 1 h at RT, and with beta-Tubulin antibody (Supplementary Table S1) at 4 °C overnight. To evaluate ECM protein expression, cells were incubated with COL1A2 and Fibronectin antibodies (Supplementary Table S1). After incubation with FITC-labelled and TRITC-labelled secondary antibodies (Supplementary Table S1), slides were mounted with Vectashield mounting medium and observed under fluorescent microscope Eclipse 600 (Nikon, Milan, Italy). NIS-Elements microscope imaging software (Nikon, version 3.22.00) was used for images acquisition.

Four images at 20× magnification were examined to determine the stress fiber score, using a five-point scoring system. The following criteria were considered: (1) little or no F-actin stress fiber formation and mainly cortical actin; (2) thin, short F-actin filaments present in at least 25% of the cell; (3) moderate F-actin stress fiber with thicker stress fibers occupying at least 50% of the cell; (4) thick and well-defined stress fibers with extensive stress fiber traversing the full width of the cell; (5) the entire cell is heavily packed with thick stress fibers, most crossing the width of the cell [45].

#### 2.9.4. Evaluation of Inflammatory and Oxidative Stress Markers

The expression of oxidative stress markers was evaluated by the DCFDA assay and Western blotting. For the DCFDA assay, cells were seeded into a black 96-well plate and treated with H_2_O_2_ and CM as already described. To detect ROS, cells were incubated with 10 μM CM-H_2_DCFDA in Hanks’ balanced salt solution (HBSS) (c6827, Invitrogen) for 45 min, before fluorescence detection on a microplate reader Infinite 200 PRO (Tecan, Männedorf, Switzerland) with excitation/emission at 485/535 nm. Results were normalized with DAPI.

For Western blotting, cells were seeded into 6-well plates and treated as described above before lysing in Denaturing Lysis Buffer (50 mM Tris-HCl, 150 mM NaCl, 1% Triton X-100, 0.1% Sodium Dodecyl Sulfate), supplemented with 1 mM PMSF, protease inhibitors (Sigma-Aldrich), and PhosStop (Roche, Basil, Switzerland). The supernatants were collected after centrifugation at 12,000× *g* for 10 min at 4 °C. Total protein amount was quantified by DC protein assay (Bio-Rad, Hercules, CA, USA). Samples (10 μg of protein for each protein extract) were prepared using NuPAGE LDS Sample Buffer 4× (Invitrogen) and NuPAGE Sample Reducing Agent 10X (Invitrogen) and fractionated in Bolt 4–12%, Bis-Tris, 1.0 mm, Mini Protein Gels (Invitrogen).

Proteins were electrophoretically transferred to 0.2 μm nitrocellulose membranes (Bio-Rad), and the obtained membranes were incubated with 5% milk in Tris-Buffered Saline containing 0.1% Tween 20 (TBS-T) to block aspecific sites. After incubation with primary antibodies at 4 °C overnight (Supplementary Table S1), the membranes were washed three times with TBS-T and subsequently incubated with the appropriate secondary antibodies (See Table S1 of the Supporting Information). Membranes were treated with Clarity Western ECL Substrate (Bio-Rad), and images were acquired with Alliance Mini HD9 (Uvitec, Cambridge, UK). Densitometric analysis was performed with Fiji software version 2.16.0 (https://imagej.net/software/fiji/downloads) accessed on 15 October 2024.

### 2.10. Statistical Analyses

The statistical analysis was performed using GraphPad Prism 10 (GraphPad Software, San Diego, CA, USA). One-way and two-way ANOVA tests were used for analyses of biological evaluation, and multiple comparisons among the groups were analyzed by Tukey’s test. Statistical significance was considered at *p* ≤ 0.05.

## 3. Results and Discussion

### 3.1. Physico-Chemical Characterization of the Films

#### 3.1.1. X-Ray Photoelectron Spectroscopy (XPS)

**Figure 1:**
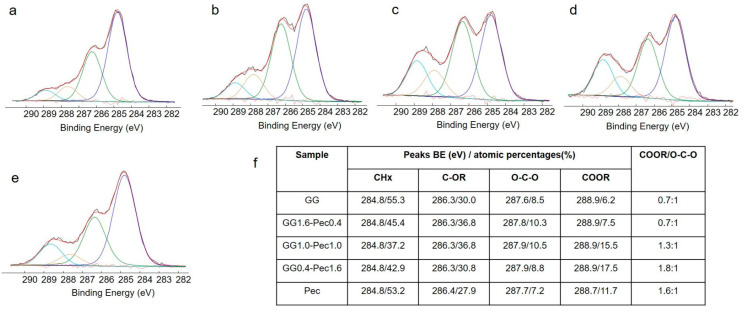
C1s high-resolution spectra and relevant curve-fitting of: (**a**) GG; (**b**) GG_1.6_-Pec_0.4_; (**c**) GG_1.0_-Pec_1.0_; (**d**) GG_0.4_-Pec_1.6_; (**e**) Pec. Peaks attribution, binding energies (BE) and atomic percentages (%) of all the C1s curve-fitting components are reported in (**f**). Maximum error on binding energy values was equal to ±0.2 eV.

An XPS analysis was carried out to investigate the surface chemical composition of the carbohydrate-based hydrogel films. Atomic percentages of the detected elements are reported in Table S2 (see Supporting Information). In Figure 1, C1s spectra and curve fitting components relevant to GG_1.6_-Pec_0.4_, GG_1.0_-Pec_1.0_, and GG_0.4_-Pec_1.6_ films (Figure 1b–d, respectively) are reported. Films based on GG (Figure 1a) and Pec (Figure 1e) were prepared and analyzed for comparison. Attributions and atomic percentages—together with the COOR/O-C-O ratios—are reported in Figure 1f. The main difference between GG and Pec films consisted in the COOR/O-C-O ratio, equal to 0.7 in GG vs. 1.6 in Pec. The blend films presented the same contributions in the C1s spectra curve fitting, carried out using four components, i.e., CHx, C-OR, O-C-O, and COOR. Glycerol, used as plasticizer, and tartaric acid, used as crosslinker, did not lead to additional peaks but to an increase in C-OR and COOR peaks. The COOR/O-C-O ratio, calculated for the blend films, showed a good correlation between this ratio and the Pec content in the blends. Shifts in the binding energies of each component were not detected in the different films, suggesting that no chemical interactions between the two carbohydrate-based polymers occurred.

#### 3.1.2. FT-IR/ATR Analysis

**Figure 2:**
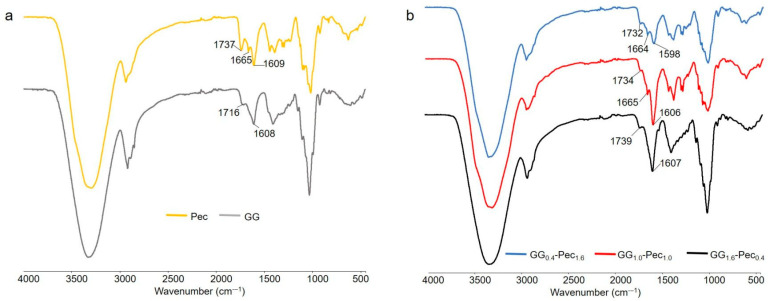
FT-IR/ATR spectra relevant to: (**a**) Pec and GG films, (**b**) GG_1.6_-Pec_0.4_, GG_1.0_-Pec_1.0_, and GG_0.4_-Pec_1.6_ films.

FT-IR analysis, carried out in ATR mode, is reported in Figure 2. Spectra of the single-component films (panel a) were acquired to clarify the main FT-IR features relevant to the blends, i.e., GG_1.6_-Pec_0.4_, GG_1.0_-Pec_1.0_, and GG_0.4_-Pec_1.6_ films (panel b). As far as the single-component films are concerned, the changes in absorption peaks observed with respect to the relevant feed powders can be mainly related to the crosslinking process. For Pec film, the peaks at 1734 and 1609 cm^−1^, relevant to the C=O stretching, were detected also in the Pec powder and can be ascribed to the methyl esterified carboxylic groups, COOCH_3_, as well as to unionized COOH groups, and to the ionized COO^-^ functionalities (asymmetric stretching), respectively. On the other hand, the presence of a peak falling at 1665 cm^−1^, not present in the FT-IR spectrum of pectin powder alone, suggests the interaction of COO^−^ groups with Ca^2+^ ions used as crosslinkers, as already observed [46].

In the GG film, in addition to the peak at 1608 cm^−1^ typical of asymmetric stretching of COO^-^ groups of GG, the peak falling at 1716 cm^−1^, absent in the relevant powder, can be assigned to the carbonyl stretching of tartaric acid crosslinker, which established ester linkages with GG, as already reported [37].

Finally, in the mixed films (panel a), the presence and the intensity of all the aforementioned peaks relating to C=O stretching vibrations were found to be present in good agreement with the proportions of Pec and GG in the three different blends.

#### 3.1.3. Thermal Characterization by TGA and DSC

**Figure 3:**
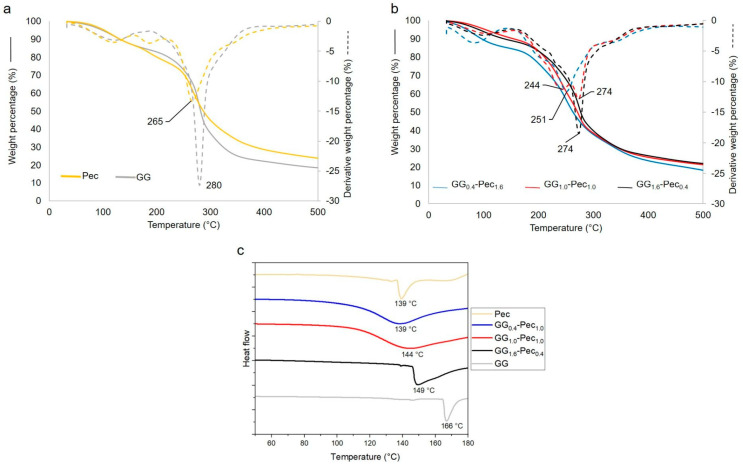
TGA (solid lines) and DTGA (dotted lines) relevant to: (**a**) Pec and GG films, (**b**) GG_1.6_-Pec_0.4_, GG_1.0_-Pec_1.0_, and GG_0.4_-Pec_1.6_ films. DSC thermograms (**c**) of Pec, GG, and the blend films (GG_1.6_-Pec_0.4_, GG_1.0_-Pec_1.0_, and GG_0.4_-Pec_1_._6_).

TGA analysis, reported in Figure 3, was carried out both on the single-component films (panel a), for comparison, and on the three blends (panel b). First, a different behavior of the obtained single-component films with respect to the polymer powders (data not reported) was observed. Indeed, the main decomposition of the Pec film occurred at higher temperatures (265 °C) than in the powder pectin (254 °C), probably due to the crosslinking process, as already reported for other polymers [47]. Similarly, GG film decomposed at a T_peak_ of 280 °C, while GG powder decomposed at 264 °C.

As far as the films are concerned, these contained water and volatiles in the 10–14% range, while single-component films contained approximately 15% water/volatiles, indicating that both the pure and the blend systems were hygroscopic. Moreover, the peak temperatures of the main decomposition of both GG and Pec polymers decreased in the blend films, suggesting a possible physical interaction between the two polymers, as already reported on other systems [48]. In the GG_0.4_-Pec_1.6_ system, the main decomposition fell at 251 °C, ascribable to Pec, and no GG peak was detected, probably falling under the shoulder of the Pec peak decomposition. In the GG_1.6_-Pec_0.4_ system, only the main decomposition peak of GG was observed at around 274 °C, while a pronounced shoulder to the left of this peak suggests that Pec decomposition occurs in that temperature range. Finally, in the GG_1.0_-Pec_1.0_ system, both the two main decomposition peaks of Pec and GG were distinctly observed, at 244 and 274 °C, respectively, being the two polymers present in equal amounts.

Finally, in Figure 3c, DSC scans relevant to pure Pec, pure GG, and the three gellan/pectin films are reported. The melting temperatures of pure Pec and GG were 139 and 166 °C, respectively. For the three blends an increase from 139 °C to 149 °C with an increase in GG amount was detected. The single melting event in the blends occurred in one large peak and this could indicate a good interaction between the two polymers, as evidenced also by TGA analysis.

### 3.2. Evaluation of Antioxidant Activity by ABTS and DPPH Assays

**Figure 4:**
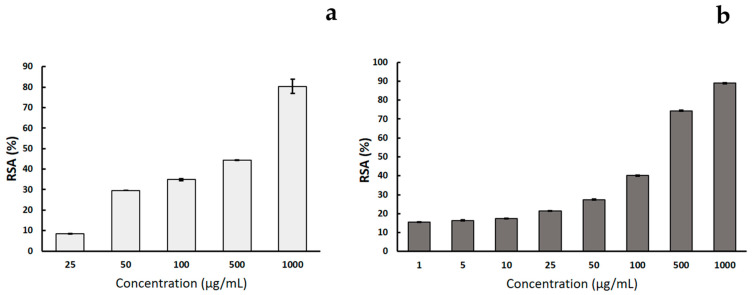
Percentage of radical scavenging activity (RSA) of CSE calculated from DPPH (**a**) and ABTS (**b**) assays. Results are reported as means ± SD of three independent experiments.

The antioxidant activity of CSE was evaluated by ABTS and DPPH assays (Figure 4). For the DPPH assay, an RSA concentration-dependent trend up to 80 ± 3% at 1 mg/mL was observed (Figure 4a). In the ABTS assay, reported in Figure 4b, a similar behavior was noted since a concentration-dependent radical scavenging activity with a maximum value of 88.90 ± 0.17% at 1 mg/mL of CSE was detected. On the other hand, for concentration values from 1 to 10 µg/mL, the scavenging activity appears almost constant between 15.00 ± 0.03% and 17.00 ± 0.03%.

**Figure 5:**
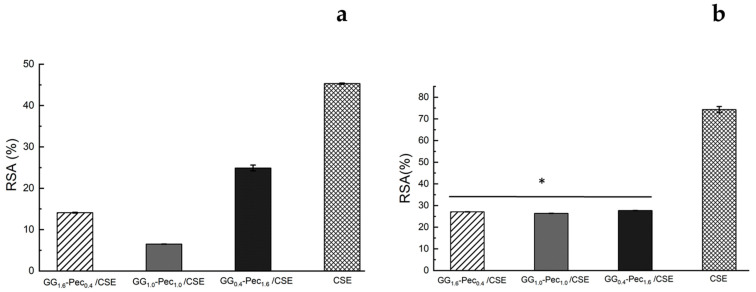
Percentage of radical scavenging activity (RSA) from DPPH (**a**) and ABTS (**b**) assays performed on CSE-loaded films. CSE solution, having a concentration equal to that embedded in the films, was also used as control in each assay. Results are reported as means ± SD of three independent experiments. Statistical analysis: * indicates no significant differences (*p* ≤ 0.05).

Figure 5 reports the antioxidant activity of the CSE-loaded films, evaluated by the DPPH and ABTS assays, and its comparison with data obtained by an amount of CSE equal to that embedded into each film. With the DPPH assay (Figure 5a), we observed a higher RSA (%), equal to 25.0 ± 0.7% for the film GG_0.4_-Pec_1.6_/CSE, followed by GG_1.6_-Pec_0.4_/CSE and GG_1.0_-Pec_1.0_/CSE with 14.1 ± 0.1% and 7.00 ± 0.04%, respectively. On the other hand, with the ABTS assay (Figure 5b) no significant differences in RSA (%) among all films were noted. However, in both assays, a reduction in the antioxidant activity of CSE-loaded films compared to the pure CSE was detected. As described in the work by Bellachioma et al. [24], CSE contains a large variety of compounds showing antioxidant capacity; therefore, the reduction in the antioxidant activity of the CSE-loaded films with respect to the CSE control could be explained considering the ability of the polymeric composite to retain part of these antioxidant compounds through intermolecular interactions, thus preventing their total release in PBS.

### 3.3. Total Polyphenol Content (TPC) and In Vitro Skin Permeation Studies

**Figure 6:**
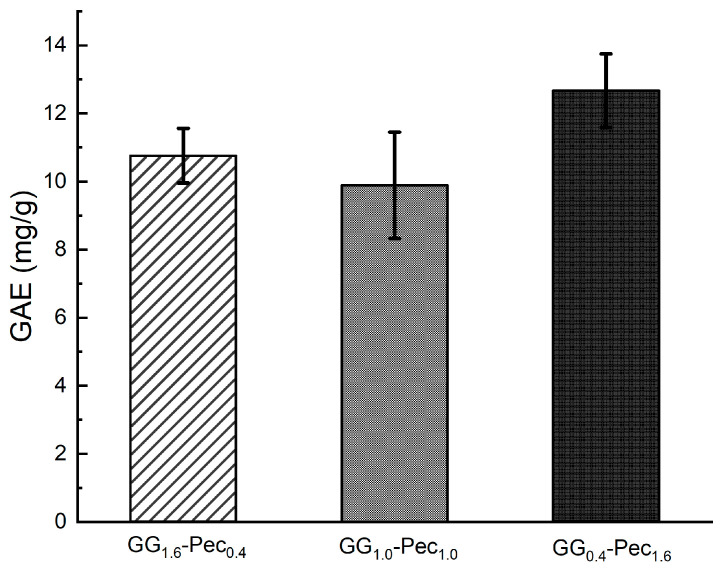
Total polyphenol content measured in CSE-loaded films after 24 h of immersion in PBS at 32 °C. Results are reported as means ± SD of three independent experiments.

The total TPC in CSE was determined using the Folin–Ciocâlteu method following the protocol described in Section 2.5 and the obtained TPC value was equal to 74 ± 3 GAE (mg/g). The TPCs of CSE-loaded films, evaluated on the solution after their immersion for 24 h, are reported in Figure 6. It can be observed that there are no significant differences among the TPC values that resulted similar for the three films and very low with respect to the value observed for CSE alone. This finding suggests that active polyphenols are likely not released from the films and are retained within the polymer backbone even after 24 h of immersion.

**Figure 7:**
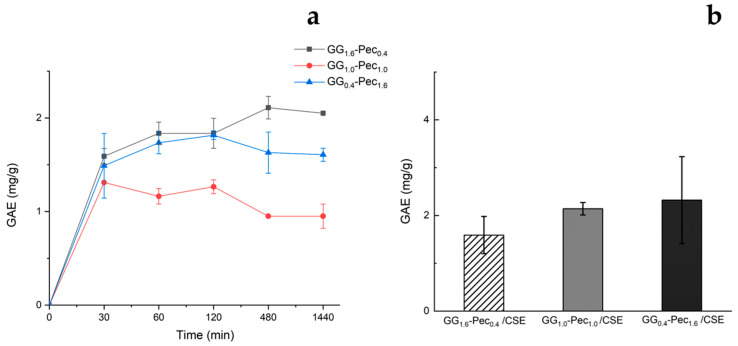
Total polyphenol content (TPC) observed after skin permeation studies through Franz diffusion cells for CSE-loaded films: (**a**) release kinetics of TPC over time; (**b**) TPC retained by StratM^®^ in contact with films for 24 h. Results are reported as means ± SD of three independent experiments.

Franz diffusion cell experiments were carried out to gain information on the transdermal release kinetics of polyphenols from CSE-loaded films. Skin permeation studies showed a release of polyphenols as a function of time for each CSE-loaded film, as reported in Figure 7a. All systems reached a plateau after about 2 h and the polyphenols concentration released after 24 h were very low, 2.05 ± 0.01, 1.00 ± 0.07, and 1.61 ± 0.13 for GG_1.6_-Pec_0.4_, GG_1.0_-Pec_1.0_, and GG_0.4_-Pec_1.6_, respectively (Figure 7a). After 24 h, TPCs were evaluated directly on the StratM^®^ membranes, which showed a small retention of polyphenols by the membranes (Figure 7b). These findings seem to be promising for films’ wound healing applications since bioactive molecules, such as polyphenols, retained in the films can induce proliferation and wound healing [49,50].

### 3.4. Hydrogel Film Swelling and Water Holding Capacity

**Figure 8:**
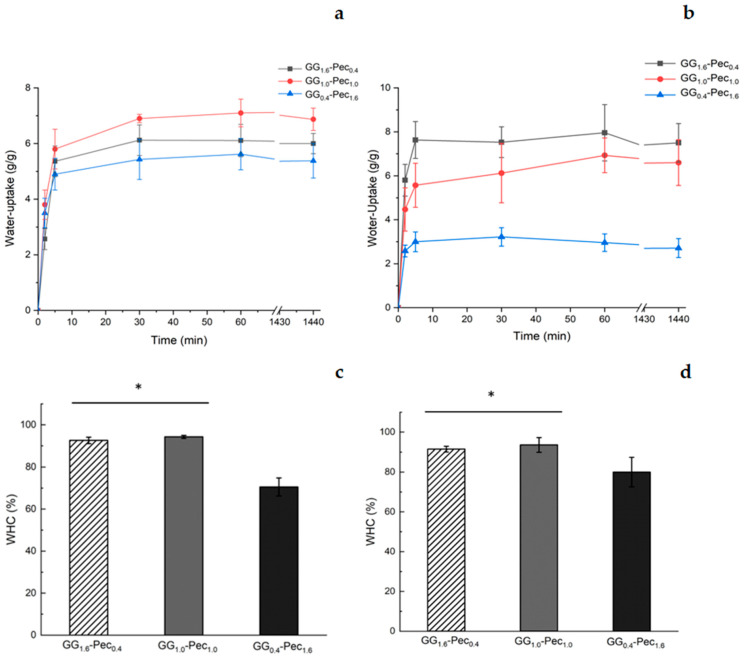
Water-uptake (*g*/*g*) (**a**,**b**) and water holding capacity (%) (**c**,**d**) for GG_1.6_-Pec_0.4_, GG_1.0_-Pec_1.0_, and GG_0.4_-Pec_1.6_ films: Water-uptake in PBS (**a**) and in SWF (**b**); WHC in PBS (**c**) and in SWF (**d**). Results are reported as means ± SD of three independent experiments. Statistical analysis: * indicates no significant differences (*p* ≤ 0.05).

Swelling and water retention performances of the hydrogels were determined through the water-uptake and water holding capacity (WHC). These parameters are very important, since a high-performance water retention suggests a good capacity to absorb an exudate generated during a skin inflammation process [51]. Tests were performed using both PBS and simulated wound fluid (SWF) (Figure 8).

In PBS, no significant differences were detected for the water-uptake profiles of the three hydrogel films (Figure 8a), while in SWF, GG_0.4_-Pec_1.6_ film showed the lowest water-uptake capacity (Figure 8b). In terms of WHC, all systems are capable of retaining water and the presence of glycerol, used as plasticizer, increased the water retention, as previously reported by Pawar et al. [52]. The best performances were displayed by GG_1.6_-Pec_0.4_ and GG_1.0_-Pec_1.0_ films showing similar behaviors in both mediums, while GG_0.4_-Pec_1.6_ film provided lower WHC values, 71 ± 3% in PBS (Figure 8c) and 80 ± 7% in SWF (Figure 8d), suggesting that the presence of more GG in the blend enhances the water retention, as already observed in a previous work on gellan/alginate films [37].

### 3.5. Indentation Test

The indentation modulus (E_IT_) and the indentation creep (C_IT_) were assessed through nanoindentation testing following the protocol described in Section 2.8. These parameters provide insights into the elastic and viscoelastic behavior of the hydrogels. E_IT_ represents the effective elastic stiffness of a material under localized deformation and C_IT_ describes the relative increase in indentation depth under a constant load.

**Figure 9:**
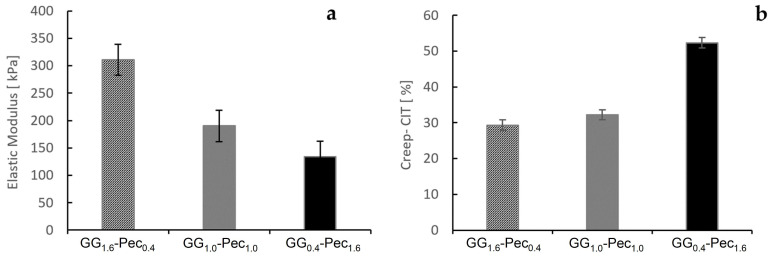
Elastic modulus (kPa) (**a**) and indentation creep (%) (**b**) for GG_1.6_-Pec_0.4_, GG_1.0_-Pec_1.0_, and GG_0.4_-Pec_1.6_ hydrogels.

The selection of the most suitable hydrogel depends on achieving an optimal balance between mechanical support (E_IT_) and compliance (C_IT_) to accommodate tissue movements. The results indicate that increasing GG content enhances the stiffness of the hydrogel films, as evidenced by the higher E_IT_ value observed for the hydrogel film GG_1.6_-Pec_0.4_ (Figure 9a). Conversely, a higher Pec proportion decreases the material’s resistance to slow deformation under sustained stress, as reflected in the elevated creep value of hydrogel film GG_0.4_-Pec_1.6_ (Figure 9b).

### 3.6. Biological Evaluations

In this work, we focused our attention on the biological effects on fibroblasts as these cells contribute to various aspects of the healing process, including extracellular matrix synthesis, immune cell recruitment, and tissue remodeling, acting as key effectors in tissue repair and regeneration [53,54]. Furthermore, the interplay between fibroblasts and the other cells involved in the healing processes is crucial and the disruptions in this crosstalk can lead to chronic wounds or fibrotic conditions, highlighting the importance of understanding these cellular interactions for successful wound healing outcomes [55].

#### 3.6.1. Film Cytocompatibility and Morphological Assessment

**Figure 10:**
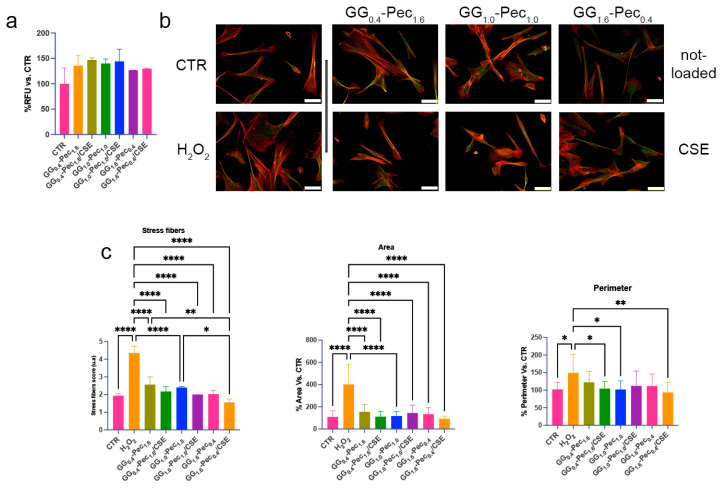
Effects of films on NhDF viability and morphology. (**a**) Histogram of cell viability after 24 h treatment with the different CM. (**b**) Representative images of the cytoskeletal staining (red: F-actin, green: β-Tubulin) and morphology observed by fluorescence microscope (20× magnification, scale bar 50 μm). (**c**) Histograms show the quantification of stress fiber formation, and the values of cell area and perimeter. (Stress fibers and area one-way ANOVA *p* ≤ 0.0001, perimeter one-way ANOVA *p* = 0.0084). (* *p* ≤ 0.05, ** *p* ≤ 0.01, **** *p* ≤ 0.0001).

To better represent a possible future scenario in skin application, cytotoxicity tests were performed treating NhDF cells with CM. No significant changes in cell viability were observed after 24 h, indicating a lack of cytotoxicity (Figure 10a).

Morphological changes in fibroblasts are important to enhance their migratory and contractile capabilities, favoring wound closure, while dysregulations, such as excessive or aberrant stress fiber formation are signs of pathological conditions [56]. In this respect, we focused on cytoskeletal protein changes in the presence of the CM. We observed an alteration in stress fiber number and diameter and a more cuboidal cell morphology after H_2_O_2_-induced damage (i.e., stressed condition) that triggered the increase in cell area and perimeter (Figure 10b,c). After incubation in CM, cells regained normal morphological parameters, with enhanced effects after treatment with media obtained from GG_0.4_-Pec_1.6_/CSE, GG_1.0_-Pec_1.0_, and GG_1.6_-Pec_0.4_/CSE (Figure 10b,c). Overall, these results show the absence of cytotoxicity from the developed films and their capability to restore regular morphological features in damaged fibroblasts, suggesting a supportive role in wound healing management.

#### 3.6.2. Evaluation of Antioxidative Properties

The first step in evaluating film effectiveness in the regulation of cell oxidative stress was the measurement of intracellular reactive oxygen species (ROS). These molecules are necessary for wound healing, but their overproduction can lead oxidative stress exacerbating tissue damage and inflammation hindering the healing process [57].

**Figure 11:**
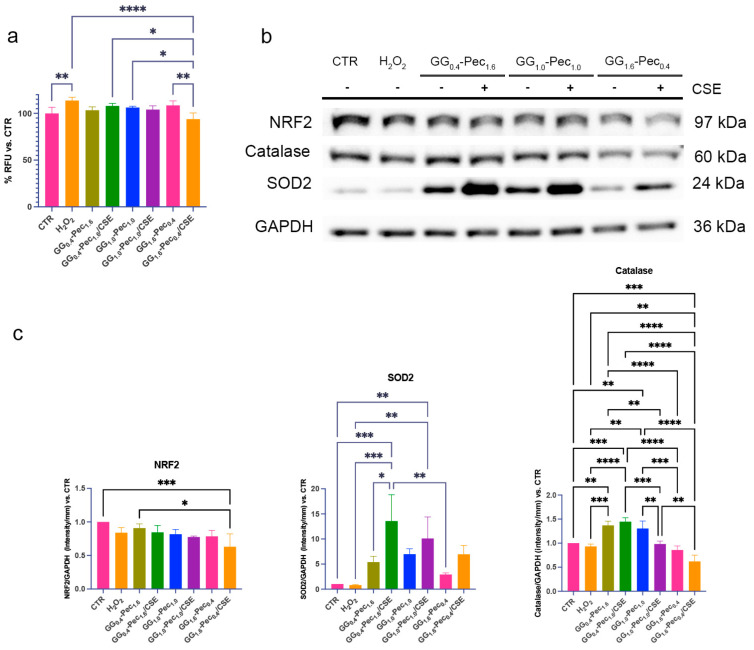
Markers for oxidative stress in NhDF cells. (**a**) Histogram of ROS levels measured with DCFDA assay (one-way ANOVA *p* = 0.0001); (**b**) representative blot for NRF2, SOD2 and Catalase; (**c**) histograms representing NRF2, SOD2 and Catalase expression (NRF2: one-way ANOVA *p* = 0.0036, SOD2: one-way ANOVA *p* = 0.0001, Catalase: one-way ANOVA *p* ≤ 0.0001) (* *p* ≤ 0.05, ** *p* ≤ 0.01, *** *p* ≤ 0.001, **** *p* ≤ 0.0001).

A slight decrease in ROS, albeit not impactful, was observed in cells after most of the CM treatments compared to H_2_O_2_-damaged cells. A significant antioxidant effect was noted for the medium from GG_1.6_-Pec_0.4_/CSE (Figure 11a), also compared to the respective unloaded film.

To counteract oxidative stress, cells implement a series of defensive strategies, such as the expression of various antioxidants and detoxifying molecules, by the activation of different pathways (i.e., NRF2) [58]. Among these, SOD2 and Catalase are two main downstream enzymes that mitigate the harmful effects of ROS during wound healing [59].

Previous studies showed that some natural compounds play a positive role against oxidative damage by activating NRF2 [60,61,62], suggesting that our CSE-loaded films could be active on this pathway.

In our systems, NhDF showed a tendency to decrease NRF2 expression in the presence of the media obtained from the CSE-loaded films, despite the concomitant increase in the expression of SOD2 and Catalase (Figure 11b,c). Compared to H_2_O_2_-stressed samples, a distinct increase in SOD2 was noted in cells treated with the CM from GG_0.4_-Pec_1.6_/CSE, with a constant decline upon increasing the amount of GG in the film formulation (Figure 11b,c). For Catalase, we observed a higher expression in the fibroblasts treated with the CM from GG_0.4_-Pec_1.6_/CSE, with the lowest enzyme level observed after incubation with the CM from GG_1.6_-Pec_0.4_ films (Figure 11b,c). Based on SOD2 and Catalase expression data, GG_0.4_-Pec_1.6_/CSE seemed the best formulation for inducing an increase in cell antioxidant enzymes. On the contrary, the DCFDA assay revealed that the highest reduction in ROS levels was detected in cells treated with the CM from GG_1.6_-Pec_0.4_/CSE, suggesting that ROS neutralization might occur by different pathways or timing as a consequence of treatment with different films.

**Figure 12:**
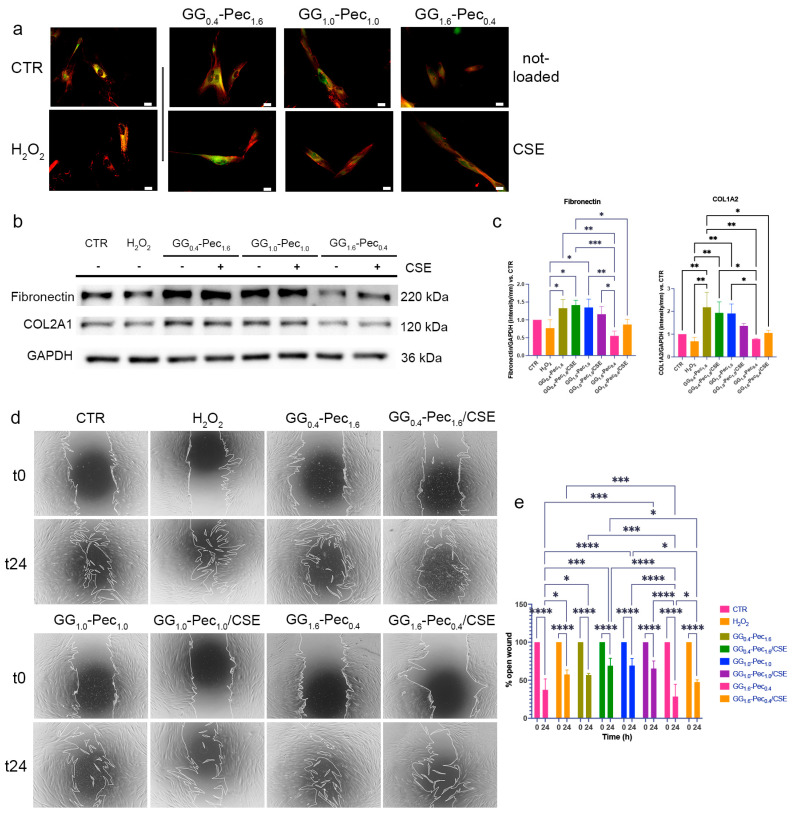
ECM protein expression and wound healing ability. (**a**) Representative images of fluorescent staining (red: Fibronectin, green: COL1A2) and (**b**) representative blot for COL1A2 and Fibronectin; (**c**) histograms for COL1A2 and Fibronectin levels estimated by Western blotting (Fibronectin: one-way ANOVA *p*= 0.0002, COL1A2 one-way ANOVA *p*= 0.0002; (**d**) representative images and (**e**) percentages of open wound (two-way ANOVA *p* = 0.0002 Time × Treatment). (* *p* ≤ 0.05, ** *p* ≤ 0.01, *** *p* ≤ 0.001, **** *p* ≤ 0.0001).

Excessive ROS production can also impair wound healing by compromising the expression and deposition of extracellular matrix (ECM) proteins, such as Type I Collagen and Fibronectin [63,64]. The treatment of cells with CM from GG_0.4_-Pec_1.6_/CSE lead to an increase in Fibronectin and COL1A2 levels, which were conversely downregulated in H_2_O_2_-stressed cells (Figure 12a–c).

Based on our data, the proposed films were able to hamper different aspects of oxidative stress in NhDF after 24 h of treatment: GG_1.6_-Pec_0.4_/CSE was the most capable in reducing intracellular ROS levels, while GG_0.4_-Pec_1.6_/CSE film was the most active in favoring the increase in the main antioxidant enzymes as well as contrasting the impairment of ECM production.

#### 3.6.3. Wound Healing Evaluation

One of the most relevant processes in wound healing is the migration of fibroblasts towards the wound bed [65,66]. To evaluate how the films may impact fibroblast migration, we performed a scratch assay, observing the healing ability after 24 h from wound induction. We observed that the CM from GG_1.6_-Pec_0.4_ films were helpful in accelerating wound closure (Figure 12d,e); therefore, we hypothesize that this formulation could have the potential to favor fibroblast migration.

## 4. Conclusions

In this study, three different blends of Pec and GG were developed and characterized as carriers for CSE. The obtained films showed suitable mechanical properties in addition to optimal swelling and water retention performances for wound dressing applications. In vitro skin permeation studies, carried out on CSE-loaded films, proved that CSE polyphenols were retained within them.

The biological data indicated that the proposed films hinder various aspects of the detrimental environment in the wound site, by modulating oxidative stress and ameliorating ECM status in NhDF, as well as favoring wound healing, suggesting a role for both the film composition and the natural extract. Overall, this study demonstrates a possible field of application for valorizing *C. sativus* tepals wasted in the saffron industry, combining the possibility of reducing agricultural waste with the development of new sustainable treatments for wound management. Indeed, by transforming agricultural by-products into valuable biomedical resources, this approach aligns with circular economy principles, promoting resource efficiency and waste minimization. Furthermore, the sustainable use of *C. sativus* tepals for wound healing applications not only supports environmental sustainability but also opens new avenues for innovative and eco-friendly biomedical solutions, potentially reducing the reliance on synthetic materials and fostering a greener healthcare industry.

## Figures and Tables

**Table 1:** Composition (expressed as weight percent to total weight of components of film) of prepared materials.

Film Code	Weight Percent (%)				
	GG	Pec	Tartaric Acid	Glycerol	CSE
GG_1.6_-Pec_0.4_	50.0	12.5	6.25	31.25	-
GG_1.0_-Pec_1.0_	31.25	31.25	6.25	31.25	-
GG_0.4_-Pec_1.6_	12.5	50.0	6.25	31.25	-
GG_1.6_-Pec_0.4_/CSE	47.6	11.9	6.0	29.7	4.8
GG_1.0_-Pec_1.0_/CSE	29.8	29.8	6.0	29.7	4.8
GG_0.4_-Pec_1.6_/CSE	11.9	47.6	6.0	29.7	4.8

**Table 2:** Instrumented indentation test parameters for each hydrogel film.

	GG_1.6_-Pec_0.4_	GG_1.0_-Pec_1.0_	GG_0.4_-Pec_1.6_
Maximal load [mN]	0.5	0.3	0.1
Loading and unloading rate [mN/min]	3	1.8	0.6
Pause at max. load [s]	10	10	10
Pause at max. load for creep determination [s]	60	60	60

## Data Availability

The original contributions presented in this study are included in the article/Supplementary Materials. Further inquiries can be directed to the corresponding author(s).

## Supplementary Materials

The following supporting information can be downloaded at: https://www.mdpi.com/article/10.3390/polym17060814/s1, Table S1: Antibodies used for the biological experiments; Table S2: Surface atomic percentages of elements detected by XPS on the investigated films.
