# Supplementary material for: Pectin/Gellan Gum Hydrogels Loaded with Crocus sativus Tepal Extract for In Situ Modulation of Pro-Inflammatory Pathways Affecting Wound Healing
Source: Polymers (Basel). 2025 Mar 20;17(6):814. doi: 10.3390/polym17060814 (PMC11945028; doi:10.3390/polym17060814)
Supplement: Supplementary file 1 [file polymers-17-00814-s001.zip › polymers-3529293-supplementary.pdf]

Pectin/Gellan Gum Hydrogels Loaded with *Crocus sativus* Tepal Extract for In Situ Modulation of Pro-Inflammatory Pathways Affecting Wound Healing

Francesco Busto [1,2], Caterina Licini [3], Stefania Cometa [4,*], Stefano Liotino [1,2], Elisabetta Damiani [5], Tiziana Bacchetti [5], Isabelle Kleider [6], Alessandra La Contana [3], Monica Mattioli-Belmonte [3,7] and Elvira De Giglio [1,2,*]

[1] Department of Chemistry, University of Bari, Via Orabona 4, 70126 Bari, Italy; francesco.busto@uniba.it (F.B.); stefano.liotino@uniba.it (S.L.)
[2] INSTM, National Consortium of Materials Science and Technology, Via G. Giusti 9, 50121 Florence, Italy
[3] Department of Clinica and Molecular Science, Università Politecnica delle Marche, via Tronto 10/a,
60126 Ancona, Italy; c.licini@staff.univpm.it (C.L.); a.lacontana@pm.univpm.it (A.L.C.); m.mattioli@staff.univpm.it (M.M.-B.)
[4] Jaber Innovation s.r.l., Via Calcutta 8, 00144 Rome, Italy
[5] Department of Life and Environmental Sciences, Polytechnic University of Marche, 60131 Ancona, Italy; e.damiani@staff.univpm.it (E.D.); t.bacchetti@staff.univpm.it (T.B.)
[6] Anton Paar TriTec SA, Vernets 6, 2035 Corcelles, Switzerland; isabelle.kleider@anton-paar.com
[7] Advanced Technology Center for Aging Research, IRCCS INRCA, Via Birarelli, 60121 Ancona, Italy
* Correspondence: stefania.cometa@jaber.it (S.C.); elvira.degiglio@uniba.it (E.D.G.); Tel.: +39-0805442016 (E.D.G.)

Table S1: Antibodies used for the biological experiments

Antibodies		
Beta-Tubulin	1:500 (IF)	T7816, Sigma-Aldrich, St. Louis, MO, USA
Fibronectin	1:100 (IF), 1:1000 (WB)	F6140, Sigma-Aldrich
COL1A2	1:100 (IF), 1:1000 (WB)	14695-1-AP, Proteintech, Manchester, UK
NRF2	1:1000 (WB)	12721, Cell Signaling, Danvers, MA, USA
Catalase	1:1000 (WB)	21260-1-AP, Proteintech
SOD2	1:250 (WB)	sc-133134, Santa Cruz Biotechnology, Dallas, TX, USA
GAPDH	1:10000 (WB)	60004-1-Ig, Proteintech
Alexa fluor 488 donkey anti-mouse	1:1000 (IF)	A21202, Thermo Fisher Scientific
Alexa Fluor 488 donkey anti-rabbit FITC	1:1000 (IF)	A21206, Thermo Fisher Scientific
Alexa fluor 555 donkey anti-mouse	1:1000 (IF)	A31570, Thermo Fisher Scientific
HRP anti-mouse	1:30000 (WB)	A90-116P, Bethyl, Montgomery, TX, USA
HRP anti-rabbit	1:10000 (WB)	A16104, Invitrogen, Waltham, MA, USA

Table S2: Surface atomic percentages of elements detected by XPS on the investigated films.

**Sample**	**Atomic Percentages(%)**			
	**C1s**	**O1s**	**N1s**	**Ca2p**
**GG**	70.6	26.5	2.6	0.2
**GG1.6-Pec0.4**	53.9	44.2	1.6	0.3
**GG1.0-Pec1.0**	54.7	44.3	0.9	0.2
**GG0.4-Pec1.6**	63.5	31.9	4.1	0.5
**Pec**	62.3	32.8	4.3	0.6
